# Diagnosis of the first few cases of a novel respiratory pathogen: the FFX-Dx protocol

**DOI:** 10.2471/BLT.25.294091

**Published:** 2025-12-02

**Authors:** Claudia M Denkinger, Jane Cunningham, Verena Faehling, Lukas E Brümmer, Kim Hanson, Richard Molenkamp, Melissa B Miller, Adrian J Marcato, David J Price, Sandra Ciesek, Emmanuel Agogo, Joseph Fitchett, Ute Ströher, Pragya D Yadav, Isabel Bergeri, Nicki L Boddington, Nira R Pollock

**Affiliations:** a Department of Infectious Disease and Tropical Medicine, University Hospital Heidelberg, German Center of Infection Research, Heidelberg, Germany.; b Health Emergencies Programme, World Health Organization, Avenue Appia 20, 1211 Geneva, Switzerland.; c Department of Medicine, University of Utah, Salt Lake City, United States of America (USA).; d Erasmus Medical Center, Rotterdam, Kingdom of the Netherlands.; e Department of Pathology and Laboratory Medicine, University of North Carolina, Chapel Hill, USA.; f Department of Infectious Diseases, University of Melbourne, Melbourne, Australia.; g Institute for Medical Virology, Goethe University, Frankfurt am Main, Germany.; h Foundation for Innovative New Diagnostics (FIND), Geneva, Switzerland.; i Institut Pasteur de Dakar, Dakar, Senegal.; j Access to Medicines and Health Products, World Health Organization, Geneva, Switzerland.; k Indian Council of Medical Research-National Institute of Virology, Pune, India.; l Department of Laboratory Medicine, Boston Children’s Hospital, Boston, USA.

For highly transmissible pathogens, accurate diagnostic testing is key to early outbreak identification, outbreak mitigation and pandemic prevention. For a novel pathogen, however, diagnostic tests are not initially available. The coronavirus disease 2019 (COVID-19) pandemic clearly demonstrated the importance, and the challenge, of rapidly developing and implementing reliable pathogen-specific diagnostic tools early in an outbreak of a novel pathogen. The diagnostic response to the spread of severe acute respiratory syndrome coronavirus 2 (SARS-CoV-2) was hindered both by the need to develop new tests on a massive scale and by inefficient approaches to test evaluation and deployment. Initial polymerase chain reaction (PCR) protocols were published within two weeks of sequence availability and the World Health Organization (WHO) was shipping kits to 159 laboratories two weeks later.^1^ Despite this accomplishment, the early response to SARS-CoV-2 was largely characterized by scarcity of diagnostic testing and few coordinated efforts to generate representative data on pathogen kinetics, the spectrum of clinical presentations, and both optimal sample types and timing of testing.^2^ These gaps hindered early efforts to prevent disease transmission and to effectively guide test utilization.

WHO has prepared a series of protocols, collectively titled the Unity Studies, that promote a harmonized approach to surveillance and rapid evidence generation for pandemic influenza viruses, SARS-CoV-2, Middle East respiratory syndrome-CoV and novel respiratory pathogens with pandemic potential (that is, pathogen X).^3^ Notably, the first protocol in the existing series, *The first few X cases and contacts (FFX) investigation template protocol for respiratory pathogens with pandemic potential,*^4^ assumes that a validated diagnostic test is already available to facilitate case detection, and does not consider that processes to ensure and optimize rapid development, evaluation and implementation of such a test need to be defined.

To fill this critical gap, a new Unity Studies protocol, *First few X cases and contacts diagnostic test evaluation for respiratory pathogens with pandemic potential: template protocol* (known as the FFX-Dx protocol) has been developed to facilitate coordinated future development, evaluation and deployment of diagnostics for a novel respiratory pathogen X. The protocol is intended to be the first of the Unity Studies to be implemented during an outbreak. An initial small group of experts developed the first draft, which was subsequently reviewed by a broad group of international diagnostic experts and additional key stakeholders and decision-makers from academia, public health and regulatory agencies. These stakeholders had led SARS-CoV-2 diagnostic test development, evaluation and implementation efforts nationally and internationally. After several rounds of review, including an in-person workshop at the University of Heidelberg, Germany,^5^ the pre-final protocol was further circulated for public consultation before it was finalized and posted on WHO’s website.^6^ The protocol has also been reviewed and revised based on input from the WHO Research Ethics Review Committee.

The protocol is designed to provide specific guidance on methods for the validation and early use of the first molecular diagnostic test for respiratory pathogen X in a rigorous but focused assessment of the first pathogen X cases and contacts at the location of an outbreak anywhere in the world. This first molecular test would need to be an in-house developed test (also known in some countries as a laboratory-developed test), given that timing of clinical and research use would need to precede receipt of any form of emergency use authorization by the relevant regulatory authority. In the protocol, the in-house developed test is assumed to be a real-time reverse transcription (RT)-PCR or PCR assay. As such, the protocol objectives were designed specifically to guide and expedite early pathogen X-specific molecular test development and deployment for clinical use and public health benefit. Optimally, if implemented early in an outbreak of pathogen X, the protocol will help to mitigate disease transmission and optimize the subsequent development and deployment of additional molecular tests, as well as point-of-care and self-tests. By providing clear steps for in-house developed test validation and generation of data to inform targeted guidance for early test use and further test development, the FFX-Dx protocol aims to aid both clinical management and the public health response for containment of an outbreak.

**Fig. 1:**
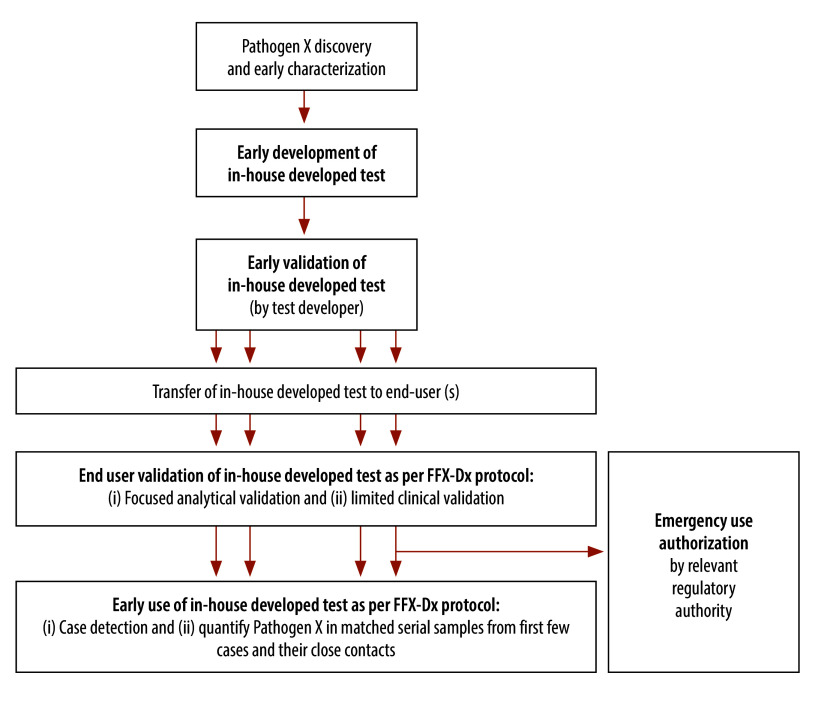
Pathway for the development and implementation of an in-house developed test for a novel pathogen X, as outlined in the FFX-Dx protocol

Note: the full name of the FFX-Dx protocol is *First few X cases and contacts diagnostic test evaluation for respiratory pathogens with pandemic potential: template protocol*.^6^

As a first step in the event of the emergence of a novel pathogen X, initial pathogen discovery and early characterization (including sequencing and culture to yield a viral or bacterial isolate) would be required and would precede the protocol. This step would be followed by early development and early validation of a molecular in-house developed test (Fig. 1) in a laboratory with staff having sufficient expertise to do this work, regardless of its distance from the outbreak site. Early test development would include work to design and develop the test for pathogen X before analytical and clinical validation, including primers, probes and an initial RT–PCR or PCR assay. This work could be done by the same laboratory involved in the discovery and/or early characterization of pathogen X or by another laboratory or laboratory consortium. The early validation phase would involve rigorous and focused analytical studies performed by the test developer to validate the test in preparation for its use for clinical (and research) testing before emergency use authorization, if required by the local regulatory authority. This early validation phase could be done before availability of clinical samples from infected patients and could be conducted by the same laboratory involved in the early development of the test or by another laboratory or laboratory consortium. Detailed considerations for the early development and early validation of the in-house developed test are presented in Annex 4 of the protocol.^6^

Following a fully completed and successful early validation performed by the test developer, a focused end-user validation would be required to put the test into clinical use for case diagnosis at the site of the outbreak (Fig. 1). This end-user validation is the subject of Part A of the protocol and would likely be performed by a nationally designated laboratory with characteristics as defined in Annex 5 of the protocol.^6^ This stage includes focused analytical validation to verify the analytical test characteristics established by the developing laboratory and a limited clinical validation, that is, prospective testing, with confirmatory testing to be performed by an independent laboratory. Upon completion of the end-user validation, the test could be made available for clinical testing. Importantly, the protocol recognizes that different jurisdictions may have alternative rules and requirements for test validation for clinical use, and that each country implementing the protocol would have to consider what additional validation might be required for emergency use authorization under its national regulatory framework.

With a validated molecular in-house developed test now available for clinical testing, the next critical steps are to define testing approaches with highest yield, optimize and refine the clinical case definition for disease X, and pre-emptively attempt to identify asymptomatic or presymptomatic infection, with the goals of preventing disease transmission and driving further test development and deployment. Part B of the FFX-Dx protocol is thus focused on early use of the validated in-house developed test to quantify pathogen X in matched (or paired) samples, that is, collected from different body sites at the same time point. These samples are collected serially over the course of infection and post-exposure to assess early pathogen X kinetics and optimal sample types for diagnostic testing in cases and contacts, including samples relevant to point-of-care and self-testing. Cumulatively, the study is designed to inform test use case(s), infection prevention and isolation duration, as well as to define optimal sample types for early molecular test deployment for clinical and reference testing and for point-of-care and/or self-test development. The workshop group defined best practice methods and materials for collection of each sample type, using knowledge gained from the COVID-19 pandemic.^2^^,^^7^ Secondary objectives of the study include preliminary estimation of key epidemiological parameters, to inform and be further studied in subsequent Unity Studies protocols.^3^

The protocol is designed to be a template that facilitates rapid deployment of in-house developed tests in the event of an outbreak, but should additionally serve as a tool or resource for national regulatory frameworks. Pandemic preparedness teams should consider early adaptation of the template protocol for in-country or national laboratories and public health authorities that are likely to take the lead in deploying the protocol. The teams should also pass the protocol through local and/or national ethics review committees. Furthermore, preparedness teams should consider how the study’s data would be used to inform targeted guidance for early test use and further test development and authorization to aid the public health response. Accordingly, the target audience for the protocol includes public health decision-makers, regulatory agencies and academic and/or research institutions, and in particular, epidemiologists and clinical laboratorians.

The expert group also recognized potential barriers to implementation of the protocol that should be addressed in advance of an emergency.^5^ These considerations include: (i) protocol implementation partners, local regulatory context and required ethics pre-approvals; (ii) funding of initial phases of test development and validation, management of intellectual property rights and possible profit-seeking behaviour by private entities; (iii) development of sample and/or organism panels (and material transfer agreements for sharing) for cross-reactivity analysis as required for early validation; (iv) navigation of regulations affecting the sharing of in-house developed test reagents and control materials between developing laboratories and end user laboratories; (v) managing supply chain logistics; (vi) facilitating data sharing; and (vii) the potential for inequities in global distribution of diagnostics.

A subgroup of experts who participated in this work are developing another template protocol for evaluation of antigen-detecting rapid diagnostic tests for a novel respiratory pathogen X.

Collectively, these efforts will facilitate the rapid generation and synthesis of high-quality evidence to guide and expedite both test development and testing policy for a novel respiratory pathogen X. Doing so will protect people’s health by expediting thoughtful deployment of tests at each stage of an outbreak response, effectively combining science, practicality, speed and equity.
